# Real-world outcomes of personalized sublingual immunotherapy for environmental allergies delivered through a telemedicine platform: a retrospective longitudinal cohort study

**DOI:** 10.3389/falgy.2026.1865860

**Published:** 2026-06-10

**Authors:** Chet Tharpe, Joshua Davidson, Kayla Mardaga, Constantine Solow, Gene Kakaulin, Tracy Kruzick, Jessica Herold, Molly Snyder, Bagrat Hakobyan

**Affiliations:** 1 Curex Inc, New York, NY, United States; 2 Pulmonary & Critical Care, BronxCare Health System, Chicago, IL, United States; 3 Medical Research & Biostatistics, Arx Sciartis LLC, Chicago, IL, United States

**Keywords:** allergic rhinitis, longitudinal outcomes, real-world evidence, SLIT, sublingual immunotherapy, telemedicine

## Abstract

**Background:**

Allergen immunotherapy (AIT) is a disease-modifying treatment for IgE-mediated respiratory allergy, but real-world use is limited by access and adherence. Telemedicine-enabled sublingual immunotherapy (SLIT) may address these barriers; long-term outcomes remain incompletely characterized.

**Methods:**

Retrospective longitudinal cohort study of adults receiving personalized SLIT via a telemedicine platform (2020–2025). Participants with ≥1 baseline and ≥1 follow-up after ≥12 months of treatment were included (*n* = 2,897). Outcomes included symptom severity, medication use, quality of life (QoL), adherence, and adverse events. Longitudinal changes were analyzed using mixed-effects models; asthma subgroup analyses were performed.

**Results:**

Mean age was 39.0 years, 52.7% were female, and 25.3% had asthma. Median follow-up was 20 months. Symptom severity decreased, with a 10.1-point reduction at 6 months and smaller subsequent declines. Clinically meaningful improvement (≥30-point reduction) increased from 28% at 12 months to 45% at 24 months; higher baseline severity predicted earlier response (HR 2.95). Medication use declined over time (OR 2.31 per 12 months), with fewer medication categories used. QoL improvement increased from 81.4% at 12–18 months to 90.7% at 30–36 months (OR 4.68 per 12 months). Self-reported adherence was high (>90% for ≥20 days/month), and discontinuation was uncommon. Adverse events were infrequent and predominantly mild (Grade I: 11.7%); no anaphylaxis or eosinophilic esophagitis was reported. Among patients with asthma, longer treatment duration was associated with improved control, reduced inhaler use, and improved QoL.

**Conclusions:**

Personalized SLIT delivered via telemedicine was associated with sustained symptom improvement, reduced medication use, high adherence, and a favorable safety profile, supporting the feasibility and scalability of this care model in routine clinical practice.

## Introduction

Allergic rhinitis (AR) and allergic airway disease affect hundreds of millions of individuals worldwide and are associated with impaired quality of life, sleep disturbance, reduced work productivity, and substantial healthcare utilization (1, 2). Conventional pharmacotherapy provides symptomatic relief but does not modify the underlying immunologic mechanisms of allergic disease. Allergen immunotherapy (AIT) remains the only disease-modifying treatment for IgE-mediated respiratory allergy, with durable benefits that may persist beyond treatment cessation (3–5).

Sublingual immunotherapy (SLIT) has emerged as a widely accepted alternative to subcutaneous immunotherapy, supported by randomized controlled trials (RCTs) and meta-analyses demonstrating efficacy for seasonal and perennial allergens with a favorable safety profile (6–9). However, despite proven efficacy under trial conditions, real-world implementation of AIT remains limited by access barriers, geographic constraints, cost, and challenges with long-term adherence (10, 11).

Telemedicine-enabled, home-administered SLIT programs aim to address these barriers by combining personalized allergen formulations with remote clinical oversight. While RCTs establish efficacy, they typically involve highly selected populations, fixed allergen compositions, and relatively short follow-up. Consequently, there is a growing need for large-scale real-world evidence to characterize longitudinal symptom trajectories, medication use, adherence, and safety in routine clinical practice over extended periods (12–14).

In this retrospective longitudinal cohort study, we evaluated real-world outcomes of a personalized SLIT program delivered through a telemedicine platform. Using repeated patient-reported assessments collected over up to five years, we examined changes in allergy symptom severity, medication use, treatment adherence, and adverse events, including a subgroup analysis of participants with asthma. By applying mixed-effects modeling, we sought to quantify both within-patient changes over time and between-patient variability, thereby describing long-term patient-reported trajectories associated with treatment exposure.

## Method

### Study design

This retrospective, longitudinal, uncontrolled, single-arm observational study evaluated real-world outcomes of a personalized, home-administered sublingual immunotherapy (SLIT) for environmental allergies delivered through a telemedicine platform (Curex). Patients received SLIT formulations (dispensed by Allergychoices pharmacy, Onalaska, WI) tailored to their environmental allergen sensitivities and were monitored remotely through periodic structured online surveys assessing symptoms, medication use, asthma-related outcomes, and adverse events. At the time of writing this manuscript, the platform had 27,257 registered users (i.e., accounts) seeking treatment for environmental and food allergies; all users underwent allergy-focused clinical evaluation and review of IgE sensitization testing, including prior skin prick testing when available and/or serum-specific IgE testing. For participants without prior testing, serum-specific IgE assessment was facilitated through ImmunoCAP™ testing during onboarding. 17,934 had received at least one treatment cycle, and 10,756 filled-out at least one on-line survey over the course of their treatment.

This study's cohort included individuals registered on the platform who initiated SLIT for environmental allergies between 2020 and 2025 and completed at least one pretreatment baseline survey and at least one post-treatment follow-up survey (n **=** 2,897). Eligible participants had confirmed IgE-mediated sensitization, established either through prior externally obtained testing submitted during onboarding (45%) or through ImmunoCAP testing facilitated through the platform (55%), received at least four SLIT prescription shipments (corresponding to 12 months of treatment), and completed the online Allergy Response Tracker (ART) survey at least twice. Survey timing criteria required completion of a baseline survey prior to or within 3 months following the first shipment, and a follow-up survey at least 12 months after treatment initiation. Each prescription shipment corresponded to a 3-month treatment cycle, such that four shipments represented a full year of therapy.

### Allergy response tracker (ART) survey

The ART survey was a structured, longitudinal instrument designed for repeated remote assessment of allergy-related outcomes in a telemedicine setting. It captured six core domains: nasal congestion, sneezing, watery eyes, symptoms affecting sleep, symptoms limiting daily activities, and overall allergy symptom control. Each item was assessed using a 5-point Likert-type scale reflecting symptom frequency or control (e.g., “rarely” to “often” for symptom items, and “complete” to “none” for overall control). Individual item responses were linearly transformed to a 0%–100% scale, with higher values indicating greater symptom burden (i.e., worse symptoms or poorer control). A composite symptom severity score was computed as the mean of all six items on the normalized 0%–100% scale, making one Likert-category improvement correspond to a 25-point reduction on ART survey symptom severity scale. A ≥ 30-point reduction threshold was selected as a pragmatic indicator of clinically meaningful improvement in lieu of a universally accepted anchor-based definition in seasonal allergy context, and consistent with the AHRQ-supported use of 30% of the maximum score as a useful threshold across symptom-based scales (15).

In addition to symptom burden, the ART survey captured allergy medication use, recorded both as the number of medication categories used (e.g., antihistamines, intranasal corticosteroids, decongestants) and as patient-reported change in use over time (ordinal categories reflecting degree of reduction). The survey also included a general quality-of-life item assessing perceived improvement relative to baseline. In participants reporting physician-diagnosed asthma, additional items assessed asthma symptom control, change in rescue inhaler use, and overall perceived change in asthma symptoms. Treatment adherence was assessed via self-reported frequency of SLIT use (days per month), allowing categorization of adherence levels over time.

Patient-reported adverse events were collected through structured survey fields and subsequently classified according to CTCAE criteria. Given the overlap between allergic symptoms and known local or systemic reactions to SLIT, some reported events (e.g., ocular, oropharyngeal, or respiratory symptoms) may reflect underlying disease activity rather than treatment-attributable effects.

All included participants provided electronic informed consent at treatment initiation permitting the use of de-identified clinical and survey data for research purposes. This retrospective analysis of de-identified data was reviewed by the WCG IRB (#20254902) and determined not to constitute human subjects research under 45 CFR 46.102(e) (1).

### Statistical method

Treatment duration was estimated as the interval between the first and last prescription shipments plus three months, corresponding to the expected duration of the final treatment cycle. Descriptive statistics were used to summarize baseline demographic and clinical characteristics. The symptom survey was designed for feasibility and repeated real-world deployment in a telemedicine context, prioritizing longitudinal consistency over formal diagnostic classification. The internal consistency of the six-item symptom survey was evaluated using Cronbach's and standardized *α*, Guttman's *λ*_6_, item-total correlations, and *α*-if-deleted statistics. Composite symptom severity scores (0%–100%) were computed as the mean of all items. We defined four cumulative endpoints based on a response defined as a ≥ 30-point absolute reduction in total symptom score from baseline; rebound was defined as any subsequent loss of this threshold after it was first achieved. The endpoints were: *response at least once*, *response with ≤1 rebound*, *response without rebound*, and *sustained response* (meeting the ≤1 rebound criterion with the ≥30-point improvement observed on ≥2 assessments), reflecting progressively stricter durability criteria. Time-to-event analyses were performed for each endpoint using Kaplan–Meier methods, with corresponding plots; analyses were stratified by baseline symptom severity, including Cox proportional hazards modeling and corresponding stratified plots.

Longitudinal changes in symptom severity were also assessed using linear mixed-effects models with participant-specific random intercepts and random slopes for time; age and sex were included as covariates in adjusted analyses. Ordinal mixed-effects (cumulative link) models with random intercepts were used to assess changes in allergy medication use and, in the asthma subgroup, changes in inhaler-use reduction, asthma symptom control, and perceived improvement. Odds ratios and 95% confidence intervals are reported. Adverse events were summarized by CTCAE grade using patient-level proportions, and time to first adverse event was analyzed using Kaplan–Meier methods with curves stratified by severity grade. We defined treatment discontinuation (non-adherence) as treatment interruption in the only available post-baseline survey, in at least 1 of 2 post-baseline surveys, or in at least 2 post-baseline surveys when 3 or more assessments were available. Treatment adherence and discontinuation were evaluated descriptively, and discontinuation risk was assessed using time-to-event analysis, including stratification by any patient-reported adverse events.

An alpha level of 0.05 was used to determine statistical relationships not due to chance. All analyses were conducted using R (version 4.5.1), with the assistance of statistical packages “*usmap”*, “*psych”*, “*lme4”*, “*lmerTest”*, “*HH”*, '*survival*' and '*survminer'.*

## Results

**Figure 1:**
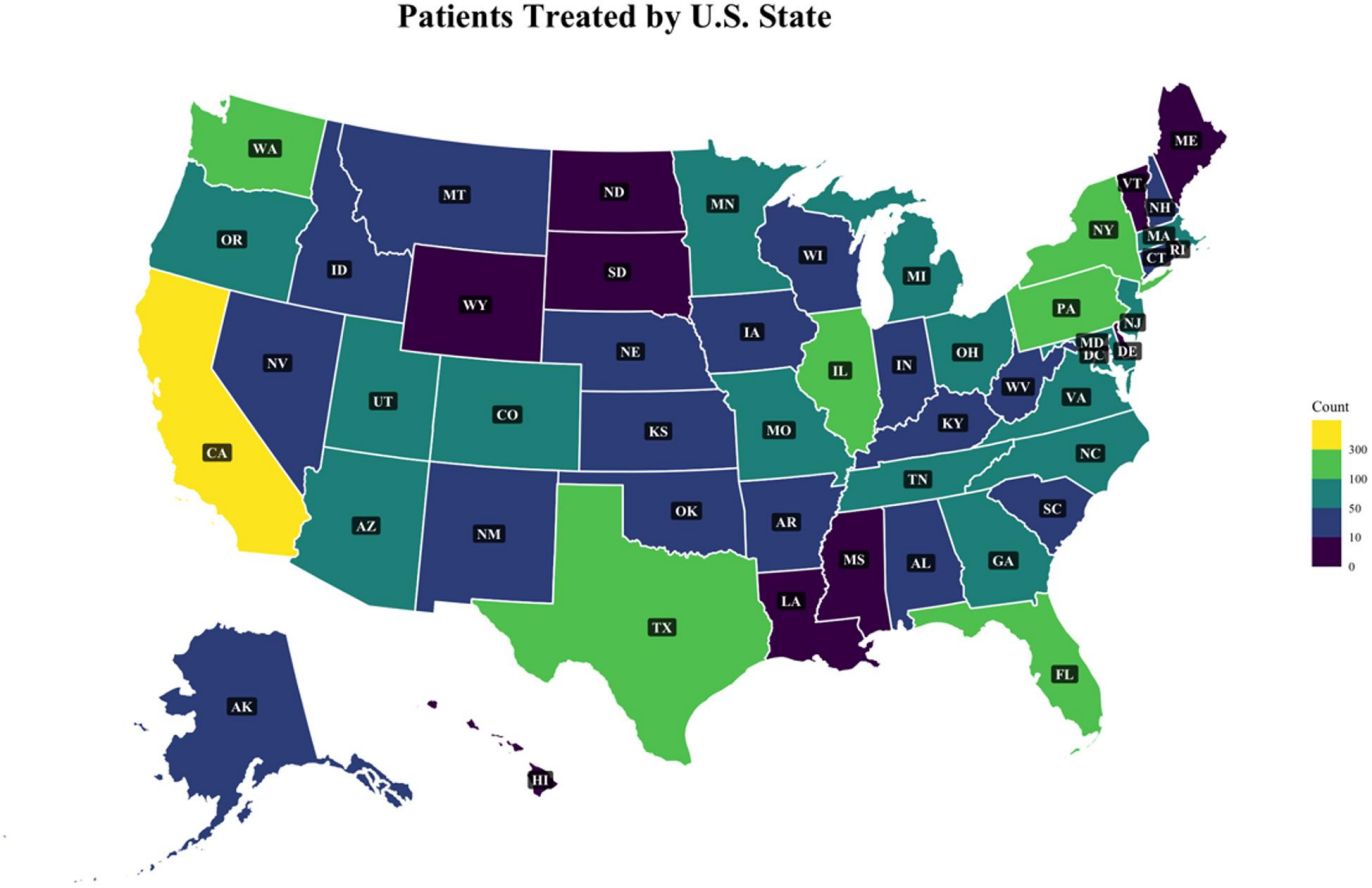
Geographic distribution of study participants.

Over the course of 2020 through 2025, a total of 2,897 participants receiving treatment for environmental allergies through a telemedicine platform were included in this analysis. Mean participant age was 39.0 (SD 12.4), and 1,527 (52.7%) were women. The geographic distribution of participants is presented in Figure 1. The treatments included sequential dilutions of environmental antigens (Table 1). The most common antigens—prescribed to more than two thirds of the sample—were derived from cat fur (76%), dust mites (75%), dog fur (72%), orchard grass (70%), and short ragweed (68%).

**Table 1:** Environmental antigens used in the study population.

Environmental antigen	Number of patients	Percent^a^
Cats	2,199	76%
Dust mites	2,182	75%
Dog	2,098	72%
Orchard grass	2,025	70%
Short ragweed	1,973	68%
Oak	1,663	57%
Bermuda grass	1,629	56%
Birch	1,595	55%
Lamb's quarters	1,502	52%
Box elder maple	1,491	51%
Cedar	1,424	49%
Ash (Fraxinus spp.)	1,326	46%
American elm	1,205	42%
Cottonwood	1,181	41%
Black walnut	1,177	41%
Alternaria (mold)	1,055	36%
American sycamore	871	30%
Mold mix I^b^	39	1%
Mesquite	26	1%
Cocklebur	9	<1%
Marsh elder	5	<1%
Mold mix II^c^	4	<1%
Olive	4	<1%

a The percentage points add up to >100% due to treatment with multiple antigens at a time.

b Comprises *Alternaria, Aspergillus, Cladosporium, Helminthosporium,* and *Penicililum* species.

c Comprises *Aureobasidium pullulans, Curvularia, Fusarium, Mucor,* and *Rhizopus* species.

**Figure 2:**
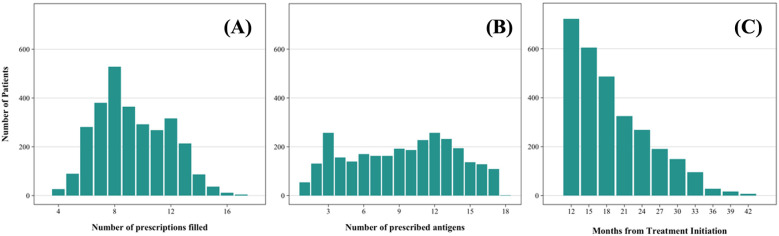
Treatment characteristics: **(A)** number of consecutive prescriptions filled; **(B)** number of prescribed antigens per treatment regimen; **(C)** last follow-up from treatment initiation.

The median treatment duration, defined as the interval between first and last shipment plus the duration of the last 3-month cycle, was 26.0 months (IQR: 22.4–34.1). During treatment, participants received a median of 9 shipments (IQR: 7–11) (Figure 2A), with a median of 10 antigens per treatment regimen (IQR: 5–13). There was a slight bimodal distribution in the number of antigens per regimen, with two distinct peaks at 3 and 12 antigens per treatment (Figure 2B). Median follow-up from treatment initiation (first shipment), defined as time to last submitted survey, was 20 months (IQR: 15.0–24.8) (Figure 2C). A subgroup of patients reported a history of clinically diagnosed asthma (*n* = 732, 25.3%). Up to two-thirds (67.3%) of participants reported using at least one allergy medication to alleviate symptoms, with second generation antihistamines (56.4%), intranasal corticosteroids (30.2%), and decongestants (19.0%) used most often (Table 2).

**Table 2:** Use of allergy medication at baseline

Medication	Number	%
Antihistamines (second generation)	1,634	56.4%
Intranasal corticosteroids	876	30.2%
Decongestants (p/o, sprays, drops, etc.)	549	19.0%
Antihistamines (first generation)	469	16.2%
Intranasal antihistamines	465	16.1%
Ocular antihistamines	285	9.8%
Long-acting beta agonists, corticosteroids, or combination inhalers	271	9.4%
Short-acting beta-agonists (i.e., rescue inhalers)	169	5.8%
Intranasal saline solutions	165	5.7%
Leukotriene receptor antagonist	154	5.3%
OTC medication not otherwise specified	143	4.9%
Topical corticosteroids	33	1.1%
Any medication	1,949	67.3%

OTC, over the counter; p/o, *per os.*

### Baseline symptoms survey

**Figure 3:**
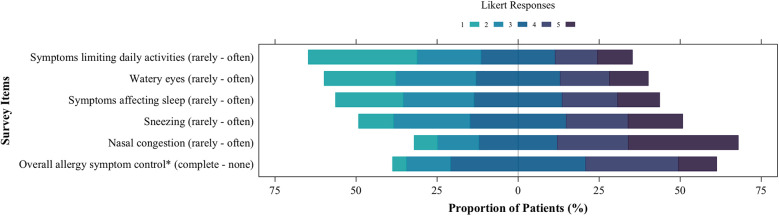
Baseline responses to general allergy symptoms survey. *Scale reversed to match direction of symptom severity.

The internal consistency of the six-item symptom survey was assessed on baseline responses (Figure 3). Cronbach's *α* was 0.78 [95% CI (0.76–0.79)], standardized *α* was 0.78, and Guttman's λ_6_ was 0.76. Inter-item correlations were moderate (average *r* = 0.36), and corrected item-total correlations ranged from 0.55 to 0.77. Reliability did not improve with removal of any item (α if deleted ranging from 0.72 to 0.77), indicating that all items contributed meaningfully to the scale. Response distributions showed full use of the 5-point range without floor or ceiling effects, and item means ranged from 2.5 to 3.6 (Table 3). Given this cohesive structure, items were combined into a composite symptom severity score (0–100).

**Table 3:** Baseline allergy symptoms survey

Survey item	Original Likert Scale (1–5)		Derived % Scale (0%–100%)	
	Mean	SD	Mean	SD
Nasal congestion (rarely—often)	3.6	(1.3)	65.6	(31.6)
Sneezing (rarely—often)	3.1	(1.2)	51.9	(30.9)
Watery eyes (rarely—often)	2.7	(1.3)	42.6	(32.3)
Symptoms affecting sleep (rarely—often)	2.8	(1.3)	44.8	(32.6)
Symptoms limiting daily activities (rarely—often)	2.5	(1.4)	36.9	(33.8)
Overall allergy symptom control^a^ (complete—none)	3.3	(1.0)	57.5	(24.7)
Total (Composite)^b^	3.0	0.9	49.9	(21.4)

a Item's scale reversed to reflect the direction of symptom severity (i.e., higher=more severe) ^b^Computed as the mean of all survey items.

At baseline, mean symptom severity was 49.9 (SD 21.4), with mean nasal congestion rated highest (65.6, SD 31.6) and symptoms limiting daily activities lowest (36.9, SD 33.8). Other symptoms showed moderate severity, including sneezing (51.9, SD 30.9), watery eyes (42.6, SD 32.3), sleep disturbance due to allergy symptoms (44.8, SD 32.6), and overall allergy symptom control (57.5, SD 24.7).

Comparison of baseline characteristics (Supplementary Table S1) across longest follow-up strata demonstrated modest differences in baseline symptom burden relative to overall score variability [estimated range in marginal means: 8.0 points, 95% CI (5.1–10.9); linear model R^2^ = 0.014], with participants in longer follow-up strata tending to have slightly lower baseline symptom scores. Older participants and those with asthma were somewhat more represented in longer follow-up strata.

### Time-to-Response analysis

**Figure 4:**
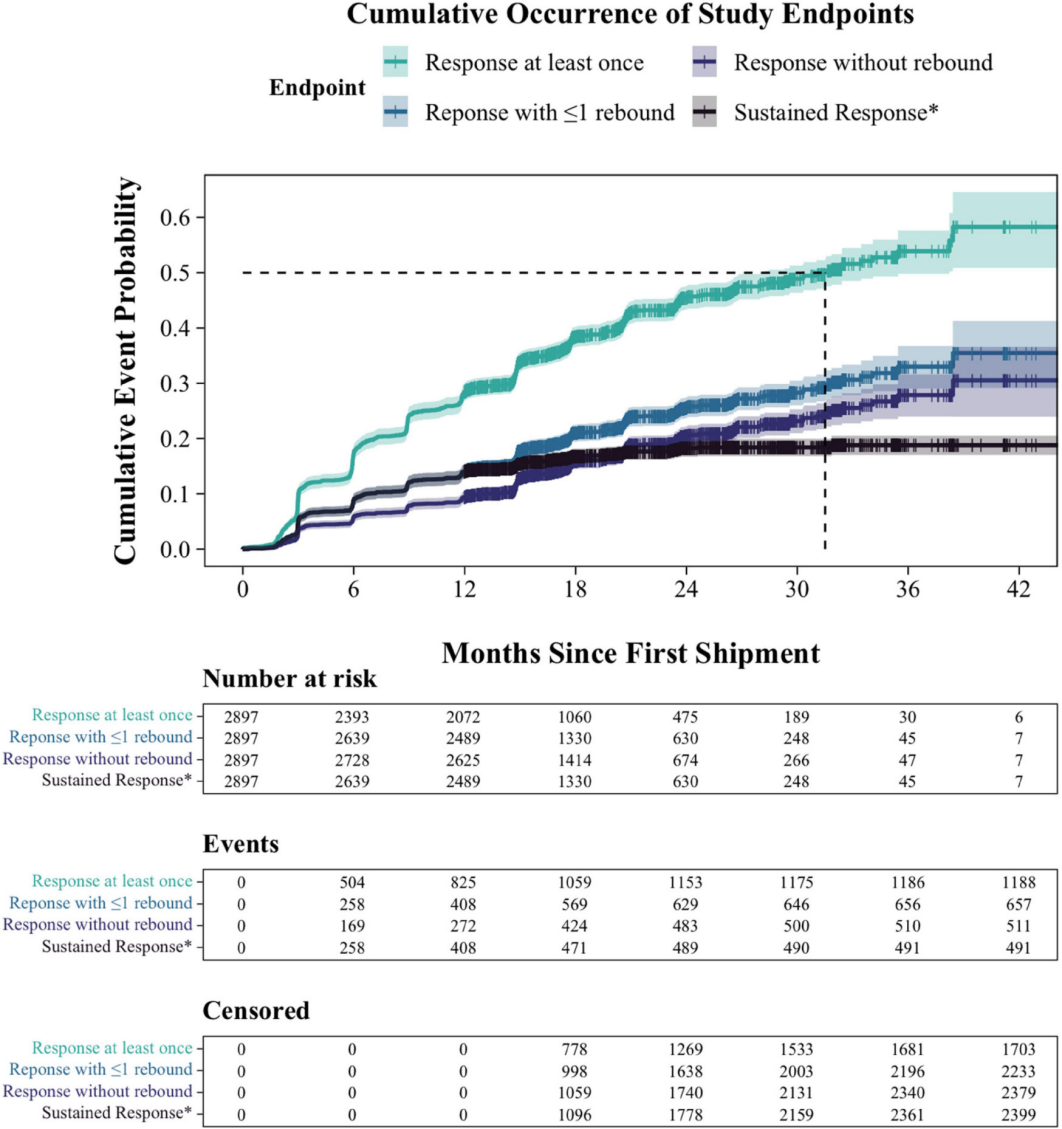
Absolute 30-point response in the study sample.^*^Four increasingly stringent definitions of response were evaluated based on a ≥ 30-point absolute reduction in total symptom score from baseline. Response at least once was defined as achieving this threshold at any follow-up assessment. Response with ≤1 rebound required that the threshold be reached and subsequently lost no more than once when ≥3 follow-up assessments were available after the first response (otherwise no rebound was allowed). Response without rebound required that the threshold be reached and maintained at all subsequent assessments without any loss of response. Sustained response required that the response definition allowing ≤1 rebound be met and that the ≥30-point improvement be observed on at least two assessments.

**Figure 5:**
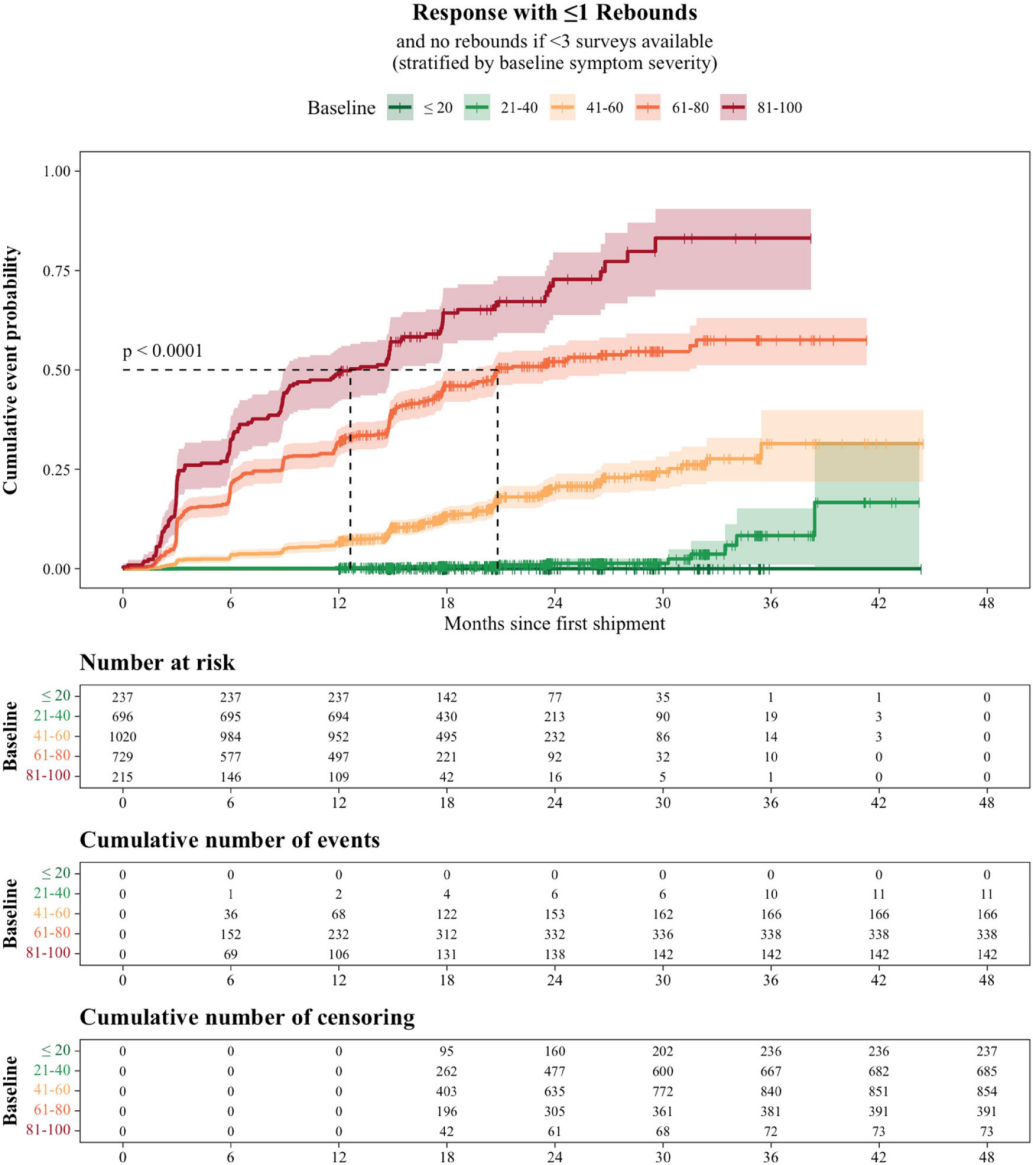
Response with ≤ 1 rebounds, stratified by baseline severity. Response with ≤ 1 rebounds was defined as a ≥ 30-point absolute reduction in total symptom score from baseline achieved at least once, with no more than one subsequent loss of response permitted when ≥3 follow-up assessments were available after the first response (otherwise no rebound was allowed). *P*-value computed from log-rank test comparing survival curves.

**Figure 6:**
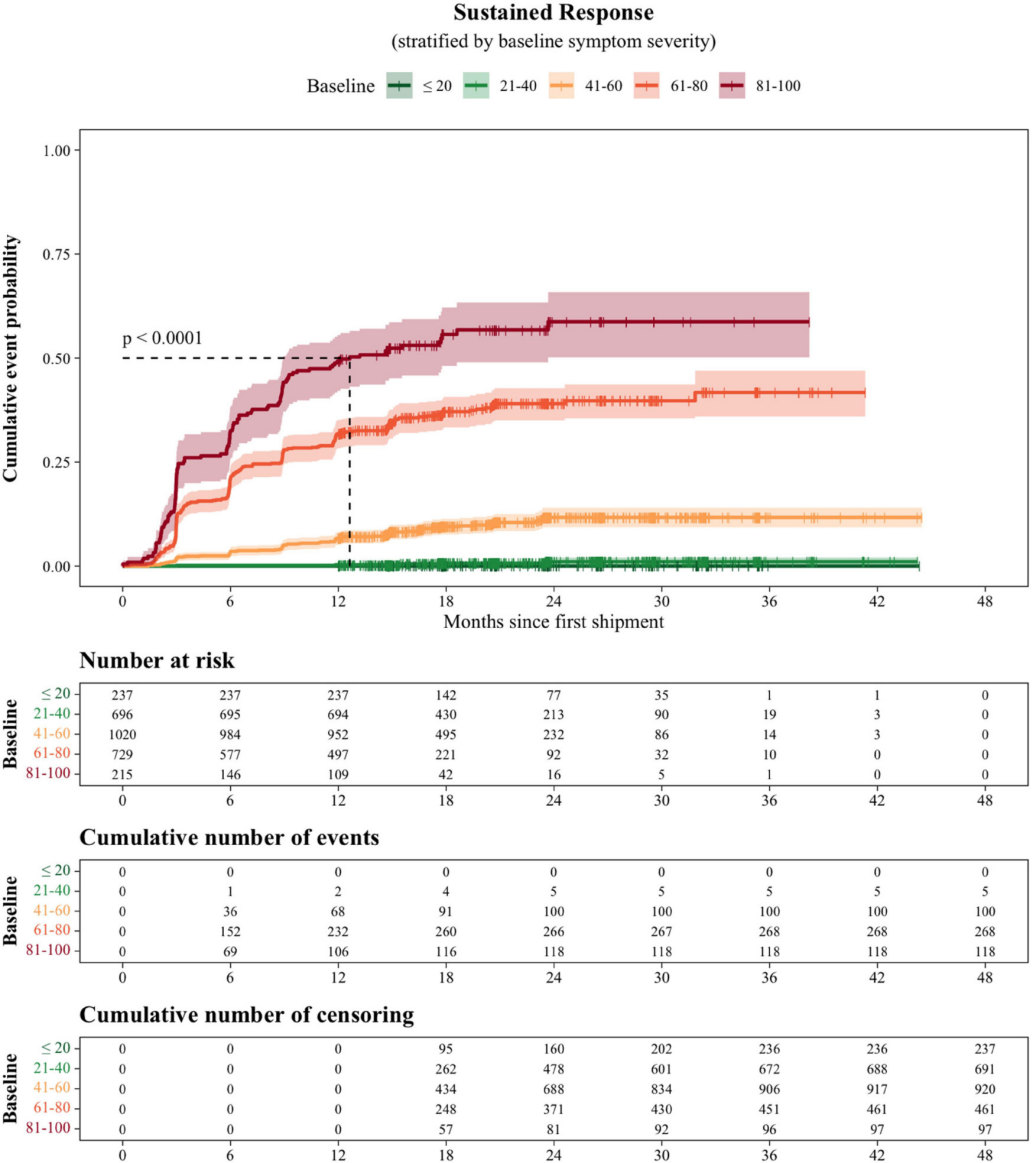
Sustained response stratified by baseline severity. Sustained response was defined as a ≥ 30-point absolute reduction in total symptom score from baseline observed on at least two assessments, with no more than one rebound (loss of response) permitted when ≥3 follow-up assessments were available after the first response, and no rebounds permitted if <3 follow-up surveys were available. *P*-value computed from log-rank test comparing survival curves.

Response, defined here as 30-point symptom reduction from baseline, increased over time across all four outcome definitions, with lower rates observed under more stringent criteria (Figure 4). At 12 months, response at least once was observed in 28% [95% CI (27–30)] (Supplementary Figure S1), response with ≤1 rebound was observed in 14% [95% CI (13–15)] (Figure 5), sustained response in 14% [95% CI (13–15)] (Figure 6), and without any rebound in 9% [95% CI (8–10)] (Supplementary Figure S2); by 24 months, these increased to 45% [95% CI (43–48)], 26% [95% CI (24–27)], 18% [95% CI (17–20)], and 20% [95% CI (19–22)], respectively. Median time to first response was 31.5 months [95% CI (28.0–38.2)]. Participants with greater baseline symptom severity achieved the ≥30-point symptom reduction earlier across all four study endpoints, with hazard ratios ranging from 2.95 to 3.57 per 20-point increase in baseline severity (*p* < 0.0001 for all; Supplementary Table S2).

### Longitudinal change in allergy symptoms

Longitudinal changes in symptom severity were evaluated using linear mixed-effects models with participant-specific random intercepts to account for within-subject correlation across repeated measures. In the unadjusted linear time model, longer follow-up duration was significantly associated with lower total symptom scores, with an average estimated reduction of 6.32 points per 12 months (*SE* = 0.18; *t* = –34.75; *p* < 0.001), suggesting a steady decline in symptom burden over time. An extended model incorporating random slopes for time and adjusting for age and sex yielded similar results, with an estimated average reduction of 6.93 points per 12 months (SE = 0.23; *p* < 0.001), suggesting a stable decline in symptom burden over time independent of demographic covariates. In this model, age was not associated with symptom severity (*p* = 0.48), whereas women reported higher scores overall (*β* = 2.83; SE = 0.52; *p* < 0.001).

However, symptom improvement followed a nonlinear trajectory, with the largest reductions occurring early after treatment initiation and diminishing over time. Furthermore, participants with higher baseline symptom severity experienced more pronounced early improvements. Supplementary Figure S3 demonstrates the distribution of the composite allergy symptom score over follow-up duration, and Supplementary Figure S4 shows the mean change from prior time interval. To capture the nonlinearity of the trajectories, longitudinal changes in symptom severity were modeled using a logarithmic transformation of time [log_(1_ _+_ _time)_]. In the latter model, at the mean baseline score, the predicted reduction from baseline to 6 months was 10.1 points [95% CI (9.52–10.6)], compared with 3.20 points from 6 to 12 months [95% CI (3.03–3.37)] and 3.38 points from 12 to 24 months [95% CI (3.20–3.56)]. Among participants with high baseline symptom burden (80 points), the corresponding predicted reductions were 28.1 points [95% CI (27.0–29.2)], 8.94 points [95% CI (8.59–9.30)], and 9.44 points [95% CI (9.07–9.82)], respectively.

### Change in allergy medication use

**Figure 7:**
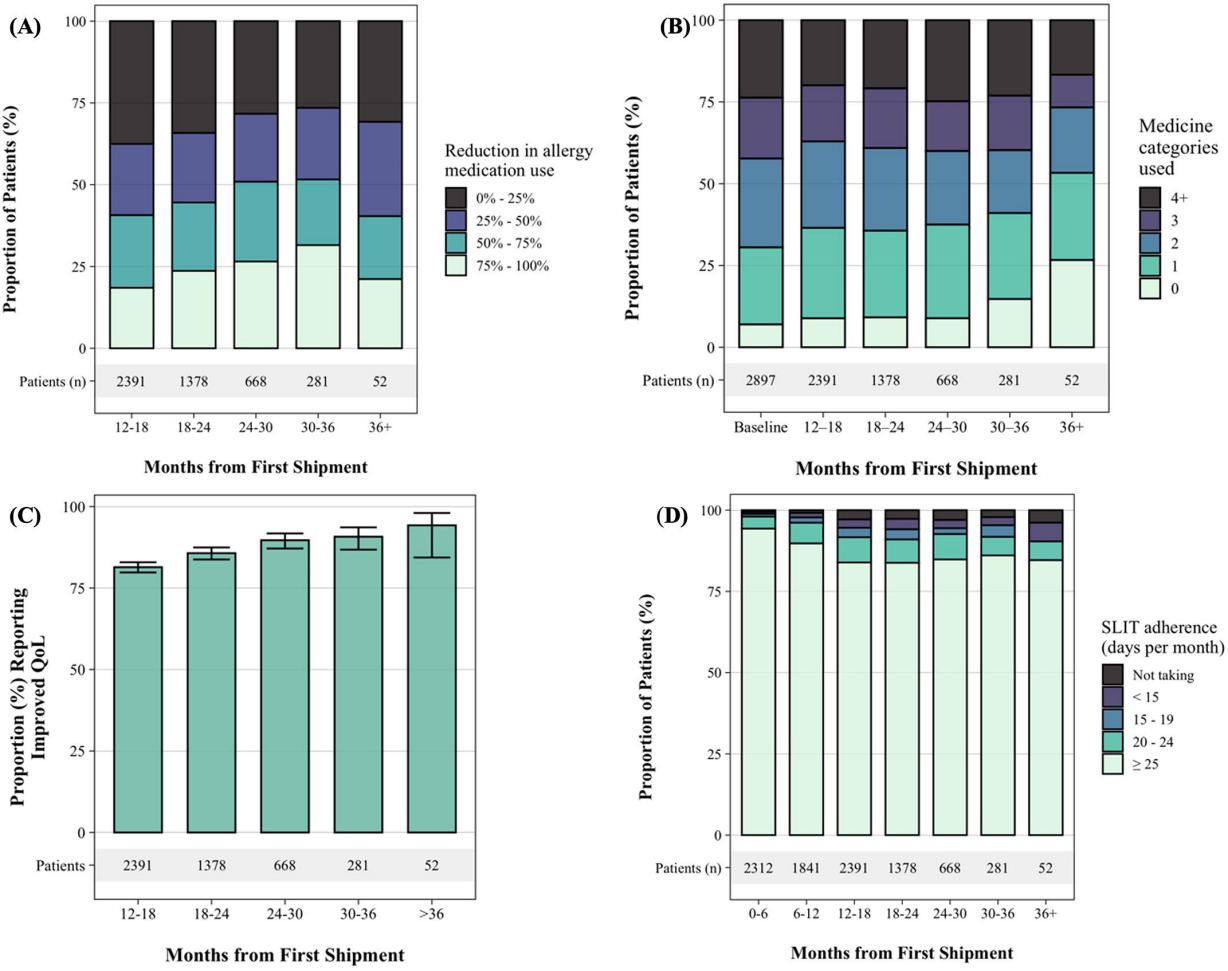
Longitudinal patterns of self-reported **(A)** reduction in overall allergy medication use; **(B)** number of allergy medication *categories* used; **(C)** improvement in quality of life; and **(D)** treatment adherence. Numbers shown in the grey strip indicate the number of patients contributing data to each interval. Error bars are 95% Wilson confidence intervals.

At 12 to 18 months, 36.8% of participants reported modest (i.e., 0%–25%) reductions in medication use, with fewer (18.2%) achieving substantial (i.e., 75%–100%) reduction. Over time, the proportion of participants with substantial reductions increased, reaching 26.2% at 24–30 months and 31.3% at 30–36 months. In parallel, the proportion reporting modest reductions declined, suggesting progressive de-escalation of medication use with longer follow-up (Figure 7A). At baseline, 7.0% of patients reported no medication use, 23.6% using one category, 27.2% using two, 18.6% using three, and 23.7% using four or more categories. Over time, the distribution shifted toward lower medication burden. The proportion of patients reporting no medication use increased from 7.0% at baseline to 8.9% at 12–18 months, 9.1% at 18–24 months, and 14.7% at 30–36 months, reaching 26.7% at ≥36 months (Figure 7B).

Ordinal mixed-effects models were used to evaluate changes in allergy medication use over time. Reductions in medication use were strongly associated with treatment duration, with each additional 12 months doubling the odds of reporting a greater reduction category [OR: 2.31, 95% CI (2.00–2.67), *p* < 0.001; Figure 7A]. Furthermore, compared with baseline, the odds of reporting a higher number of medication categories used reduced as early as 12–18 months [OR 0.71, 95% CI (0.62–0.82)], with further reductions observed at 24–30 months [OR 0.64, 95% CI (0.51–0.82)], 30–36 months [OR 0.54, 95% CI (0.36–0.80)], and ≥36 months [OR 0.10, 95% CI (0.04–0.24)] (Figure 7B).

### Improvement in quality of life

The proportion of patients reporting improved quality of life remained consistently above 80% throughout follow-up, increasing over time from 81.4% [95% CI (79.8–82.9)] at 12–18 months to 85.7% [95% CI (83.8–87.5)] at 18–24 months and 89.7% [95% CI (87.1–91.8)] at 24–30 months, with further increases observed at 30–36 months [90.7%, 95% CI (86.8–93.6)] and ≥36 months [94.2%, 95% CI (84.4–98.0)]. The estimates at later time points must be interpreted with caution given the smaller number of observations (Figure 7C). In a mixed-effects logistic model, each additional 12 months of treatment was associated with a 4.68-fold increase in the odds of reporting improved quality of life [OR: 4.68, 95% CI (3.40–6.44), *p* < 0.001].

### Treatment adherence

Treatment adherence remained high throughout follow-up, with >90% of patients consistently reporting SLIT use on ≥20 days per month and >80% reporting use on ≥25 days per month across all time intervals. At 0–6 months, 94.3% of patients reported ≥25 days of use, decreasing modestly to 89.8% at 6–12 months and 83.9% at 12–18 months, and remaining stable thereafter (83.8% at 18–24 months, 84.8% at 24–30 months, 86.1% at 30–36 months, and 84.6% at ≥36 months). Lower adherence categories, including infrequent (on <15 days per month) or no use, remained relatively uncommon throughout follow-up, though their proportion increased slightly, from <3% during the first year to approximately 5% to 6% at most later intervals, reaching 9.6% at ≥36 months. (Figure 7D).

A total of 42 patients met criteria for treatment discontinuation over the course of follow-up. Among these, adverse events were reported in 6 patients. All events were grade I in severity and included eye pruritus (*n* = 3), mouth pruritus (*n* = 1), urticaria (*n* = 1), and non-urticarial rash (*n* = 1). No higher-grade adverse events were observed in association with treatment discontinuation. In time-to-event analysis, treatment discontinuation was not associated with reporting of adverse events (*p* = 0.81, Supplementary Figure S6).

### Adverse events

Adverse events were uncommon and predominantly mild. Overall, 11.7% of patients reported at least one Grade I event, most frequently ocular and cutaneous symptoms, including eye pruritus (3.24%), cutaneous rash (2.14%), generalized pruritus (1.69%), headache (1.52%), and throat irritation (1.42%). Other Grade I events, including gastrointestinal, respiratory, and neurologic symptoms, each occurred in ≤1% of patients (Table 4). Grade II events were less frequent (3.73% overall), most commonly edema of the mouth, tongue, or lips (0.62%), asthma exacerbation (0.55%), dyspnea (0.52%), and wheezing (0.38%), with all other events occurring in ≤0.3% of patients. Higher-grade events were rare: Grade III adverse events occurred in 0.83% of patients and were distributed across multiple systems without a dominant pattern, including isolated cases of ocular symptoms, respiratory events, gastrointestinal symptoms, and hypersensitivity reactions such as urticaria and angioedema. Notably, no Grade IV or V events were observed, and no cases of eosinophilic esophagitis or anaphylaxis were reported.

**Table 4:** Adverse events observed in the study cohort.

Adverse Event^a^	Grade I		Grade II		Grade III	
	n	%	n	%	n	%
^b^Eye pruritus	94	3.24%	4	0.14%	5	0.17%
^b^Eye edema	13	0.45%	2	0.07%	0	0.00%
^b^Throat irritation	41	1.42%	6	0.21%	0	0.00%
^b^Oral pruritus (itching of the oral mucosa)	27	0.93%	1	0.03%	0	0.00%
^b^Edema of mouth, tongue, or lips	3	0.10%	18	0.62%	1	0.03%
Oral Pain	6	0.21%	0	0.00%	0	0.00%
Oral ulceration	1	0.03%	0	0.00%	0	0.00%
^b^Generalized pruritus	49	1.69%	9	0.31%	3	0.10%
^b^Urticaria	28	0.97%	3	0.10%	4	0.14%
^b^Generalized angioedema	0	0.00%	0	0.00%	2	0.07%
^b^Cutaneous rash (non-urticarial)	62	2.14%	5	0.17%	4	0.14%
^b^Dyspnea	1	0.03%	15	0.52%	2	0.07%
^b^Cough	31	1.07%	6	0.21%	4	0.14%
^b^Wheezing	1	0.03%	11	0.38%	0	0.00%
^b^Asthma exacerbation	0	0.00%	16	0.55%	2	0.07%
New-onset asthma	0	0.00%	6	0.21%	0	0.00%
Dysphagia	3	0.10%	0	0.00%	1	0.03%
Nausea	6	0.21%	0	0.00%	3	0.10%
Vomiting	1	0.03%	0	0.00%	1	0.03%
Abdominal pain	3	0.10%	0	0.00%	1	0.03%
Diarrhea	2	0.07%	0	0.00%	1	0.03%
Eosinophilic Esophagitis	0	0.00%	0	0.00%	0	0.00%
Headache	44	1.52%	5	0.17%	3	0.10%
Tinnitus	2	0.07%	1	0.03%	1	0.03%
Dizziness	0	0.00%	1	0.03%	1	0.03%
Fatigue	19	0.66%	4	0.14%	4	0.14%
Anaphylaxis	—		—		0	0.00%
Any adverse event	339	11.7%	108	3.73%	24	0.83%

a Severity classified per Common Terminology Criteria for Adverse Events (CTCAE) v5.0.

Percentages represent the proportion of patients experiencing at least one event of the specified grade (*N* *=* *2,897*). Patients may report multiple adverse events; totals are not additive.

Dashes indicate inapplicable severity grade.

No grade IV or V adverse events were observed.

b Reported adverse events may overlap with allergic disease manifestations and may not be fully distinguishable from treatment-related effects. Reported AE rates may therefore reflect, in part, underlying symptom burden*.*

Time-to-event analysis demonstrated that patients remained largely adverse event-free over follow-up, with events accumulating gradually over time. At 12 months, the probability of remaining adverse event-free was 92.7% [95% CI (91.7–93.6)] overall, including 94.4% [95% CI (93.6–95.3)], 98.0% [95% CI (97.5–98.5)], and 99.5% [95% CI (99.3–99.8)] for Grade I, II, and III events, respectively. By 24 months, the probability of remaining adverse event–free was 82.8% [95% CI (81.1–84.5)] overall, with corresponding estimates of 86.5% [95% CI (84.9–88.0)], 95.6% [95% CI (94.7–96.5)], and 99.0% [95% CI (98.5–99.4)] across increasing severity grades. Median time to first adverse event was not reached for any severity grade. Detailed Kaplan–Meier estimates are provided in Supplementary Figure 5.

### Subgroup analysis: patients with asthma

**Figure 8:**
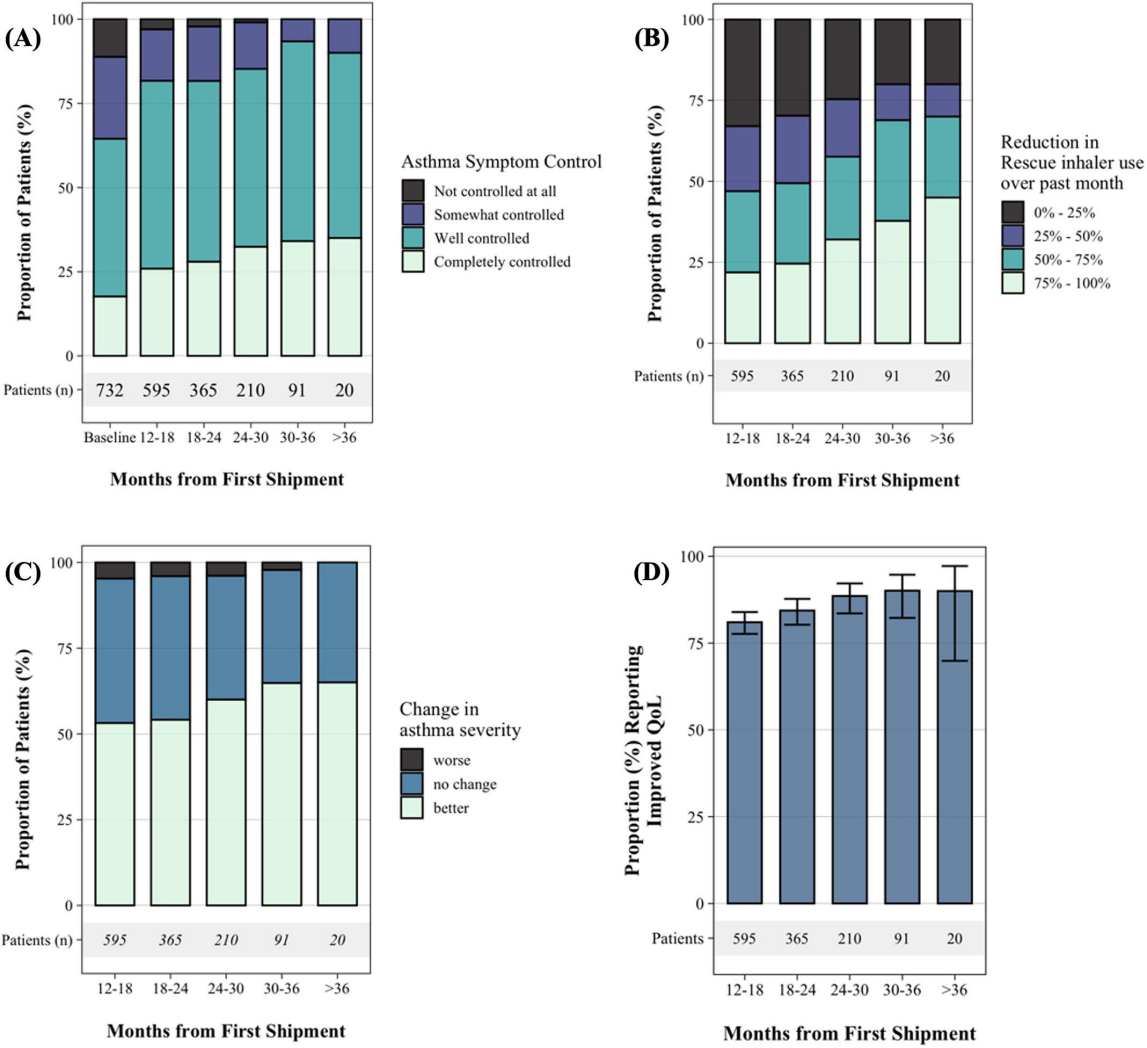
Findings from the follow-up of the subgroup with asthma, demonstrating **(A)** improved symptom control, **(B)** reduced rescue inhaler use, **(C)** overall subjective change in asthma severity compared to pre-SLIT baseline, and **(D)** proportion reporting improved quality of life (QoL) over time. Error bars are 95% Wilson confidence intervals.

In the subgroup of participants who reported physician-diagnosed asthma at baseline (*n* = 732, 25.3%), 11.2% reported asthma symptoms as not controlled at all and 17.6% as completely controlled. Over time, the proportion of patients with uncontrolled symptoms decreased substantially, falling to 3.0% at 12–18 months, and not observed later at 30–36 months of follow-up. In parallel, the proportion of patients reporting completely controlled symptoms increased to 25.9% at 12–18 months, with continued increases observed at later time points (27.9% at 18–24 months, 32.4% at 24–30 months, 34.1% at 30–36 months, and 35.0% at ≥36 months; Figure 8A). Patients also reported greater reductions in rescue inhaler use over time. The proportion of patients reporting at least 50% reduction increased from 47.0% at 12 to 18 months to 49.5% at 18 to 24 months, 57.6% at 24 to 30 months, 68.9% at 30 to 36 months, and 70.0% at ≥36 months (Figure 8B). The proportion of patients reporting overall improvement in asthma symptoms slightly increased over time, from 53.2% at 12 to 18 months to 54.1% at 18 to 24 months, 60.0% at 24 to 30 months, 64.8% at 30 to 36 months, and 65.0% at ≥36 months. In contrast, the proportion of patients reporting worsening symptoms remained low and decreased over time, from 4.7% at 12 to 18 months to 4.0% at 18 to 24 months, 3.9% at 24 to 30 months, and 2.2% at 30 to 36 months, with no patients reporting worsening at ≥36 months (Figure 8C). These changes were accompanied by increasing proportions of self-reported improvement in quality of life compared to pre-SLIT baseline: from 81.0% [95% CI (77.7–84.0)] at 12 to 18 months to 90.1% [95% CI (82.3–94.7)] at 30 to 36 months (Figure 8D).

In ordinal mixed-effects models, each additional 12 months of treatment was associated with a 2.72-fold increase in the odds of reporting a higher symptom control category [OR: 2.72, 95% CI (2.45–3.03), *p* < 0.001], a 2.31-fold increase in the odds of reporting a greater inhaler reduction category [OR: 2.31, 95% CI (1.79–3.00), *p* < 0.001], and a 2.16-fold increase in the odds of reporting a higher overall symptom improvement category [OR: 2.16, 95% CI (1.49–3.12), *p* < 0.001]. In a mixed-effects logistic model, each additional 12 months of treatment was associated with a 4.39-fold increase in the odds of reporting improved overall quality of life [OR: 4.39, 95% CI (2.38–8.11), *p* < 0.001].

## Discussion

This large real-world longitudinal analysis found that personalized SLIT delivered via a telemedicine platform was associated with longitudinal improvements in allergic symptoms, reduced reliance on allergy medications, high adherence, and a favorable safety profile. The magnitude and trajectory of symptom improvement observed in this cohort were consistent with a clinically meaningful change and broadly aligned with prior randomized trials and observational SLIT studies using validated symptom measures (6–8, 16). Importantly, this study extends prior work by demonstrating consistent longitudinal outcome patterns across multiple patient-reported measures within a large-scale telemedicine-based personalized SLIT program. Reductions in allergy medication use paralleled improvements in symptom burden. Progressive decreases in pharmacotherapy requirements have been consistently reported in patients responding to allergen immunotherapy (17, 18). The present findings add to this body of evidence by demonstrating sustained reductions in medication use over time in a real-world cohort, with temporal patterns that align with observed improvements in symptom burden.

In patients with comorbid asthma, improvements in symptom control, reductions in rescue inhaler use, and favorable shifts in patient-reported outcomes were observed over time. These findings are consistent with prior evidence demonstrating that allergen immunotherapy may improve asthma outcomes and reduce exacerbation risk (19–21). This study adds longitudinal real-world evidence consistent with these associations across multiple asthma-related endpoints.

The comparative effectiveness of single- and multi-allergen allergen immunotherapy (ATI) in polysensitized individuals remains incompletely defined. Existing studies comparing these approaches have generally been small and heterogeneous and have not consistently demonstrated superior outcomes with multi-allergen ATI (22–24). Consequently, current practice guidelines remain cautious in preferentially endorsing multi-allergen ATI over single-allergen approaches, particularly in the context of standardized FDA- and EMA-approved formulations (25), while also recognizing that multi-allergen ATI remains the predominant practice pattern throughout North America (26). In the present study, most participants were polysensitized and received multi-allergen SLIT formulations (median 10 antigens per regimen, IQR 7–11), whereas only a small minority received single-allergen treatment (1.9%; *n* = 55). Given the limited number of single-allergen recipients, direct comparison between treatment approaches was not pursued. Accordingly, the longitudinal outcomes observed in this study should primarily be interpreted as real-world evidence reflecting contemporary North American multi-allergen approach to SLIT and may not be generalizable to international practice contexts.

Selection and survivor bias also warrant consideration. Inclusion required completion of at least one baseline and one follow-up survey, resulting in the exclusion of participants with incomplete survey data. Comparison across longest follow-up strata (Supplementary Table S1) demonstrated only modest differences in baseline symptom burden, with follow-up duration strata accounting for no more than 1.4% of baseline symptom score variance. Older participants and those with asthma were slightly more represented among longer-followed strata, potentially reflecting greater long-term engagement with chronic disease management. Participants who remained engaged with long-term follow-up may also represent a subset with greater treatment adherence, persistence, or perceived benefit, potentially contributing to overestimation of longitudinal improvements observed in routine practice. Additionally, estimates at ≥36 months should be interpreted as exploratory, given the smaller number of participants contributing data at this later follow-up interval.

However, improvement was evident early and persisted across follow-up durations, suggesting that findings are not solely driven by selective retention. Seasonal variability is a well-recognized challenge in longitudinal allergy research (27, 28). Due to asynchronous enrollment, geographic heterogeneity, individualized allergen profiles, and inconsistent alignment of surveys with peak exposure periods, a unified seasonality adjustment was not feasible. Over extended follow-up, seasonal effects are expected to average out, particularly in cohorts with repeated measures spanning multiple seasons (29).

Adherence and discontinuation were defined using conservative criteria designed to distinguish transient interruptions from sustained cessation. Treatment adherence remained high across all time intervals, and discontinuation was uncommon. Notably, discontinuation was rarely associated with adverse events, all of which were mild, consistent with prior reports indicating that tolerability is not the primary driver of SLIT discontinuation in real-world practice (10, 30). These findings provide additional real-world evidence for adherence in a telemedicine-based care setting. The observed adverse events were infrequent and predominantly mild (Table 4), consistent with those reported in large SLIT trials and post-marketing surveillance studies (7, 8, 31). Importantly, no cases of eosinophilic esophagitis or anaphylactic reactions were reported, consistent with the overall safety of the intervention. While the voluntary nature of reporting may result in underestimation of mild events, the rarity of higher-grade reactions is consistent with the established safety profile of SLIT, potentially extending to telemedicine-delivered care settings.

The ART survey used in this study was developed pragmatically to balance clinical relevance with feasibility in a telemedicine context. Although the survey has not undergone formal external validation against standardized instruments, the Likert-scale symptom severity items used to derive the composite symptom score incorporate ordinal patient-reported symptom domains and functional impairment measures (Table 3), paralleling established symptom-based allergy scales such as TNSS (32). Nevertheless, direct comparisons between the outcomes observed in this study and published SLIT trials utilizing externally validated instruments should be interpreted with caution.

### Limitations

The absence of a concurrent control group and randomization are inherent limitations of this retrospective observational study. As a single-arm analysis without an untreated comparator, improvements over time may partially reflect regression to the mean or other unmeasured factors. Accordingly, the findings should be interpreted as descriptive of observed symptom trajectories rather than causal estimates of treatment effect. The within-person longitudinal design partially mitigates confounding by allowing each participant to serve as their own control. Mixed-effects models accounted for repeated measures and baseline heterogeneity, enabling estimation of population-level trends while preserving individual variability. However, residual confounding cannot be excluded. All outcomes were self-reported through voluntary surveys, introducing the possibility of reporting, recall, and social desirability bias (33). This may have influenced estimates of symptomatic improvement, medication use reduction, and treatment adherence. Adverse events, particularly mild and transient reactions, may also be under-reported in this setting. The symptom assessment instrument was developed for pragmatic use in a telemedicine context and has not undergone external validation.

Missing data were addressed using a combination of last observation carried forward (LOCF) logic for descriptive summaries and likelihood-based mixed-effects and cumulative link mixed models for inferential analyses, which allow inclusion of all *available* observations and accommodate unbalanced longitudinal follow-up without requiring explicit imputation. Inspection of follow-up patterns did not suggest systematic dropout driven by observed symptom severity. Nonetheless, bias cannot be fully excluded if missingness depended on unmeasured outcomes or time-varying factors not captured in the models.

This study reflects outcomes observed within a commercially operated real-world care model. The study was conducted using data collected through the Curex telemedicine platform; several authors were involved in the clinical operation and development of the platform and were affiliated with Curex Inc. during study conduct and manuscript preparation; and statistical analysis was performed independently by a third party engaged by Curex Inc. for biostatistical support. While these affiliations and relationships are disclosed in accordance with journal requirements, the potential biases associated with the commercial context are acknowledged.

## Conclusion

In this large real-world cohort, personalized SLIT delivered via a telemedicine platform was associated with sustained improvements in allergic symptoms, reduced medication use, high adherence, and a favorable safety profile over long-term follow-up. Improvements were observed across multiple outcome domains, including symptom burden, medication reliance, quality of life, and asthma-related endpoints. Although causal inference is limited by the observational single-arm design, similar longitudinal patterns were observed across multiple endpoints and analytic approaches. These results complement existing clinical trial evidence and are consistent with real-world feasibility and scalability of telemedicine-enabled SLIT in routine clinical practice.

## Data Availability

The raw data supporting the conclusions of this article will be made available by the authors, without undue reservation.
