# Supplementary material for: Real-world outcomes of personalized sublingual immunotherapy for environmental allergies delivered through a telemedicine platform: a retrospective longitudinal cohort study
Source: Front Allergy. 2026 Jun 10;7:1865860. doi: 10.3389/falgy.2026.1865860 (PMC13290930; doi:10.3389/falgy.2026.1865860)
Supplement: Supplementary file 1 [file Table1.pdf]

**Supplemental Table 1.** Baseline characteristics stratified by longest observed follow-up duration.

| Longest follow-up (months) | | Symptom Score | | Female | | Age | | Asthma | |
|---|---|---|---|---|---|---|---|---|---|
| | **Group N** | **Mean** | **SD** | **n** | **%** | **Mean** | **SD** | **n** | **%** |
| *12-18* | 1328 | 52.3 | (21.3) | 721 | 54% | 38.3 | (12.9) | 279 | 21% |
| *18-24* | 812 | 48.8 | (21.1) | 425 | 52% | 38.9 | (11.7) | 164 | 20% |
| *24-30* | 460 | 47.8 | (21.2) | 226 | 49% | 39.5 | (12.0) | 107 | 24% |
| *30-36* | 245 | 44.3 | (21.7) | 126 | 52% | 40.9 | (12.7) | 59 | 24% |
| *>36* | 52 | 48.9 | (19.3) | 29 | 57% | 45.6 | (12.7) | 18 | 35% |
