# Supplementary material for: Real-world outcomes of personalized sublingual immunotherapy for environmental allergies delivered through a telemedicine platform: a retrospective longitudinal cohort study
Source: Front Allergy. 2026 Jun 10;7:1865860. doi: 10.3389/falgy.2026.1865860 (PMC13290930; doi:10.3389/falgy.2026.1865860)
Supplement: Supplementary file 2 [file Table2.pdf]

Supplemental Table 2. Time to Response and Effect of Baseline Symptom Severity Across Response Definitions.

Outcome*	Time Since First Shipment (Months)	Cumulative Response Estimate [95% CI]		Baseline Symptom Score HR† [95% CI]		*p*-value†
Response achieved at least once	12	0.28	[0.27–0.30]	2.95	[2.78–3.14]	<0.0001
	24	0.45	[0.43–0.48]			
	36	0.54	[0.50–0.58]			
Response with ≤1 rebound	12	0.14	[0.13–0.15]	3.38	[3.10–3.68]	<0.0001
	24	0.26	[0.24–0.27]			
	36	0.33	[0.29–0.37]			
Sustained response (≥2 assessments, ≤1 rebound)	12	0.14	[0.13–0.15]	3.57	[3.22–3.95]	<0.0001
	24	0.18	[0.17–0.20]			
	36	0.19	[0.17–0.21]			
Response without rebound	12	0.09	[0.08–0.10]	2.99	[2.72–3.29]	<0.0001
	24	0.20	[0.19–0.22]			
	36	0.28	[0.24–0.32]			

*Response defined as ≥30 percentage point reduction in total symptom score; rebound defined as subsequent loss of this threshold after initial response.

†Hazard ratio per 20-point increase in baseline symptom score; values represent relative rates of achieving response over time (Cox proportional hazards models); *p*-values correspond to the Wald test for the baseline symptom score coefficient in each model.
