# Supplementary material for: Real-world outcomes of personalized sublingual immunotherapy for environmental allergies delivered through a telemedicine platform: a retrospective longitudinal cohort study
Source: Front Allergy. 2026 Jun 10;7:1865860. doi: 10.3389/falgy.2026.1865860 (PMC13290930; doi:10.3389/falgy.2026.1865860)
Supplement: Supplementary file 3 [file Image1.pdf]

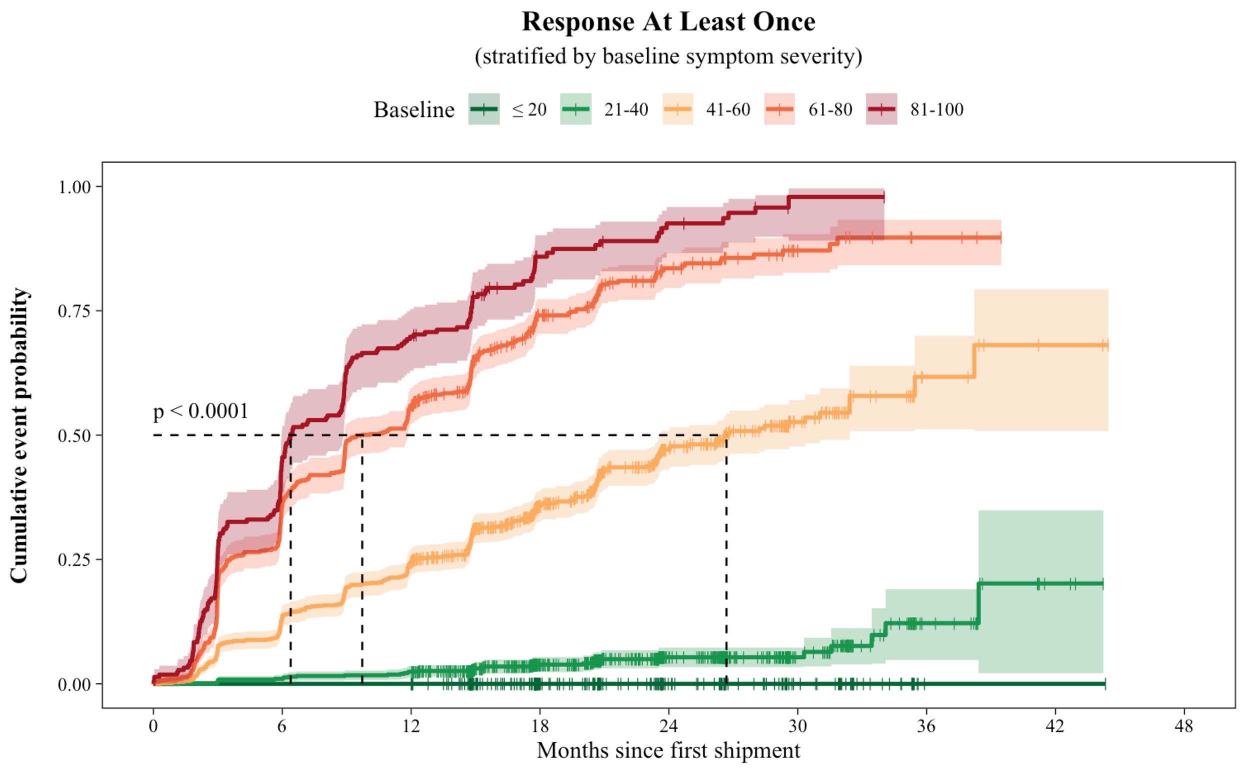

Number at risk

Baseline	0	6	12	18	24	30	36	42	48
≤ 20	237	237	237	142	77	35	1	1	0
21-40	696	689	679	415	205	88	19	3	0
41-60	1020	881	770	372	150	54	7	2	0
61-80	729	468	321	113	35	11	3	0	0
81-100	215	118	65	18	8	1	0	0	0

Cumulative number of events

Baseline	0	6	12	18	24	30	36	42	48
≤ 20	0	0	0	0	0	0	0	0	0
21-40	0	7	17	25	30	30	34	35	35
41-60	0	139	250	343	393	405	410	411	411
61-80	0	261	408	513	544	550	552	552	552
81-100	0	97	150	178	186	190	190	190	190

Cumulative number of censoring

Baseline	0	6	12	18	24	30	36	42	48
≤ 20	0	0	0	95	160	202	236	236	237
21-40	0	0	0	256	461	578	643	658	661
41-60	0	0	0	305	477	561	603	607	609
61-80	0	0	0	103	150	168	174	177	177
81-100	0	0	0	19	21	24	25	25	25

Supplemental Figure 1. Response at least once during follow-up, stratified by baseline symptom severity. The endpoint was defined as achieving a 30-point symptom reduction from baseline at any follow-up assessment. P-value computed from log-rank test comparing survival curves.
