# Supplementary material for: Real-world outcomes of personalized sublingual immunotherapy for environmental allergies delivered through a telemedicine platform: a retrospective longitudinal cohort study
Source: Front Allergy. 2026 Jun 10;7:1865860. doi: 10.3389/falgy.2026.1865860 (PMC13290930; doi:10.3389/falgy.2026.1865860)
Supplement: Supplementary file 4 [file Image2.pdf]

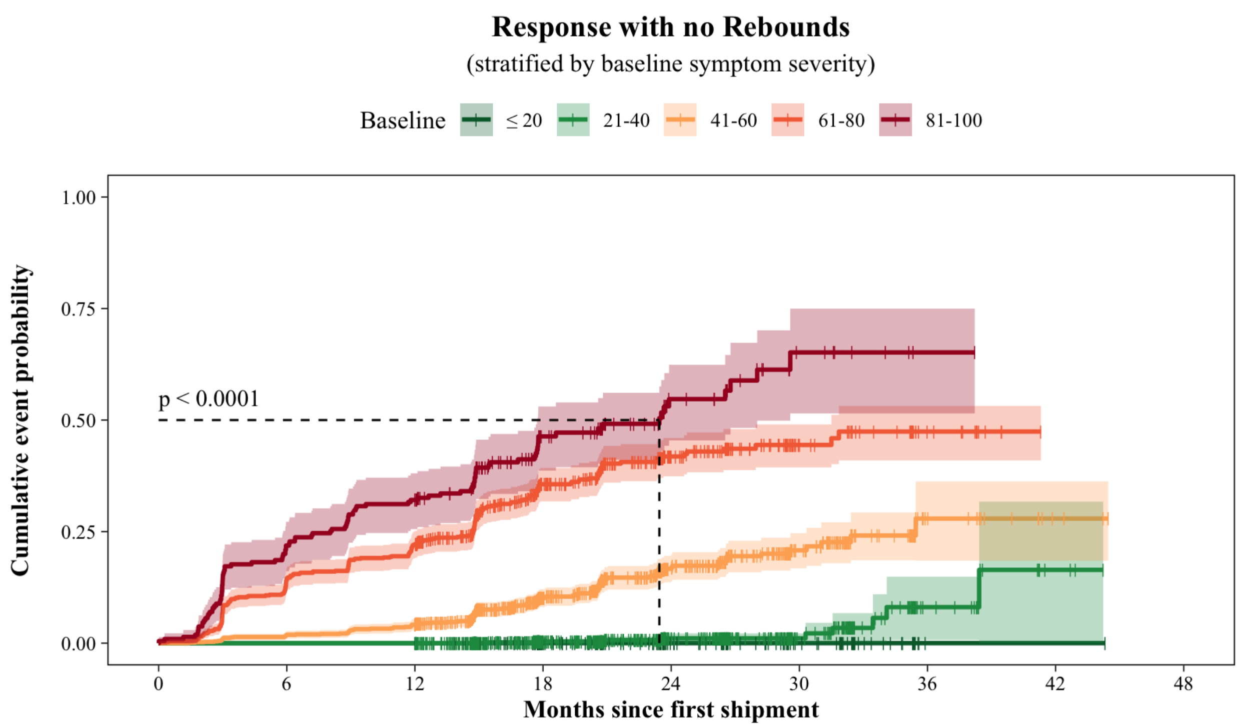

Number at risk

Baseline	0	6	12	18	24	30	36	42	48
≤ 20	237	237	237	142	77	35	1	1	0
21-40	696	696	696	431	214	91	19	3	0
41-60	1020	1001	978	516	245	92	14	3	0
61-80	729	626	568	260	111	40	12	0	0
81-100	215	168	146	65	27	8	1	0	0

Cumulative number of events

Baseline	0	6	12	18	24	30	36	42	48
≤ 20	0	0	0	0	0	0	0	0	0
21-40	0	0	0	2	4	4	8	9	9
41-60	0	19	42	91	121	130	134	134	134
61-80	0	103	161	238	258	262	264	264	264
81-100	0	47	69	93	100	104	104	104	104

Cumulative number of censoring

Baseline	0	6	12	18	24	30	36	42	48
≤ 20	0	0	0	95	160	202	236	236	237
21-40	0	0	0	263	478	601	669	684	687
41-60	0	0	0	413	654	798	872	883	886
61-80	0	0	0	231	360	427	453	465	465
81-100	0	0	0	57	88	103	110	111	111

Supplemental Figure 2. Response with no observed rebounds, stratified by baseline symptom severity. The endpoint criterion required that the 30-point absolute symptom reduction from baseline be reached and subsequently lost no more than once when ≥3 follow-up assessments were available after the first response (otherwise no rebound was allowed). P-value is computed from log-rank test comparing survival curves.
