# Supplementary material for: Real-world outcomes of personalized sublingual immunotherapy for environmental allergies delivered through a telemedicine platform: a retrospective longitudinal cohort study
Source: Front Allergy. 2026 Jun 10;7:1865860. doi: 10.3389/falgy.2026.1865860 (PMC13290930; doi:10.3389/falgy.2026.1865860)
Supplement: Supplementary file 5 [file Image3.pdf]

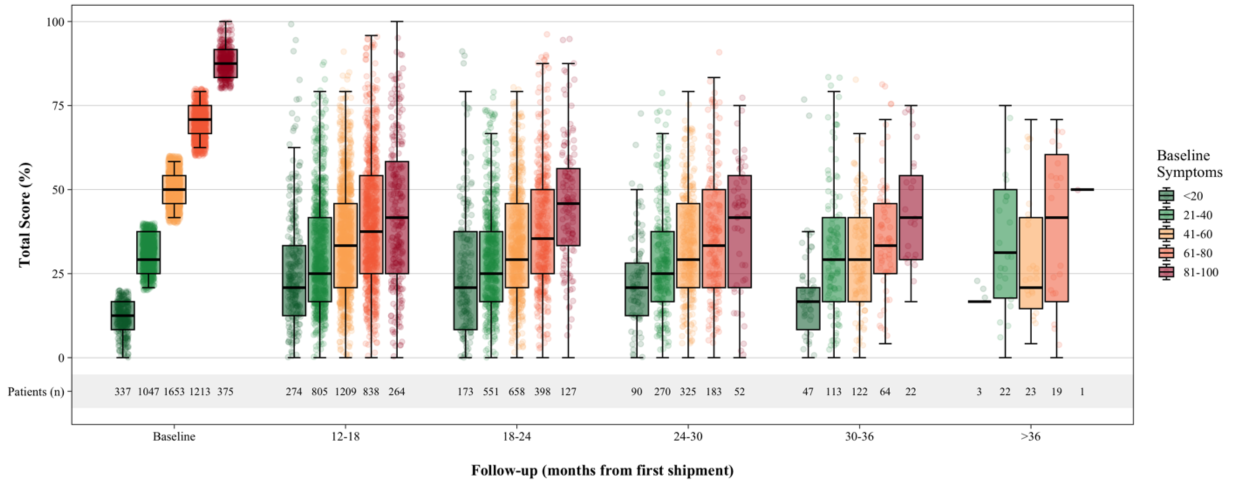

Supplemental Figure 3. Distribution of Total Symptom Scores Over Time Stratified by Baseline Symptom Severity. Points represent individual survey responses. Numbers below the x-axis indicate the number of patients contributing data at each timepoint.
