# Supplementary material for: Real-world outcomes of personalized sublingual immunotherapy for environmental allergies delivered through a telemedicine platform: a retrospective longitudinal cohort study
Source: Front Allergy. 2026 Jun 10;7:1865860. doi: 10.3389/falgy.2026.1865860 (PMC13290930; doi:10.3389/falgy.2026.1865860)
Supplement: Supplementary file 6 [file Image4.pdf]

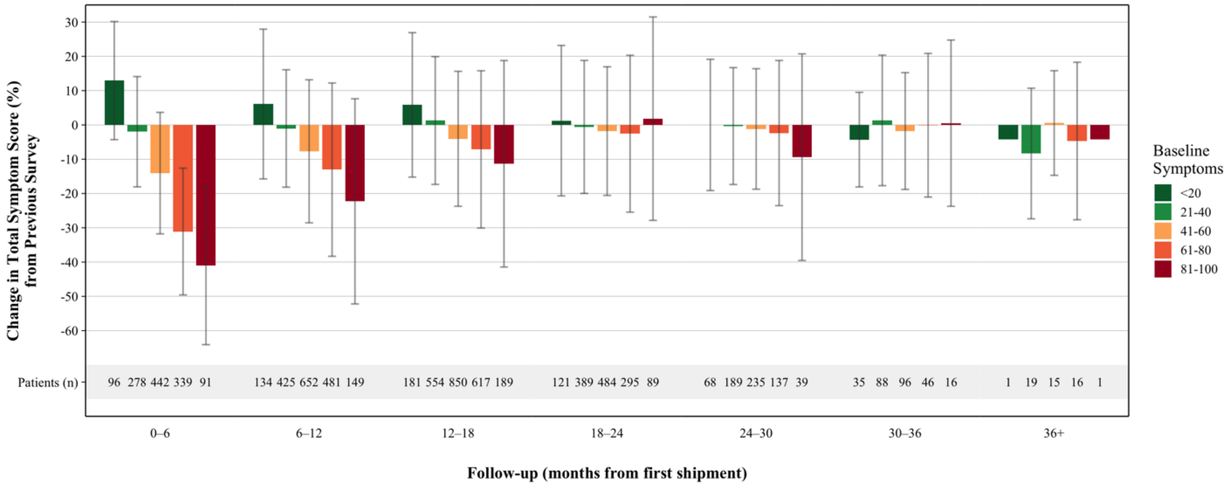

Supplemental Figure 4. Change in total symptom score relative to the most recent prior completed survey across follow-up intervals, stratified by baseline symptom severity (columns are means and error bars are SD).
