# Supplementary material for: Real-world outcomes of personalized sublingual immunotherapy for environmental allergies delivered through a telemedicine platform: a retrospective longitudinal cohort study
Source: Front Allergy. 2026 Jun 10;7:1865860. doi: 10.3389/falgy.2026.1865860 (PMC13290930; doi:10.3389/falgy.2026.1865860)
Supplement: Supplementary file 7 [file Image5.pdf]

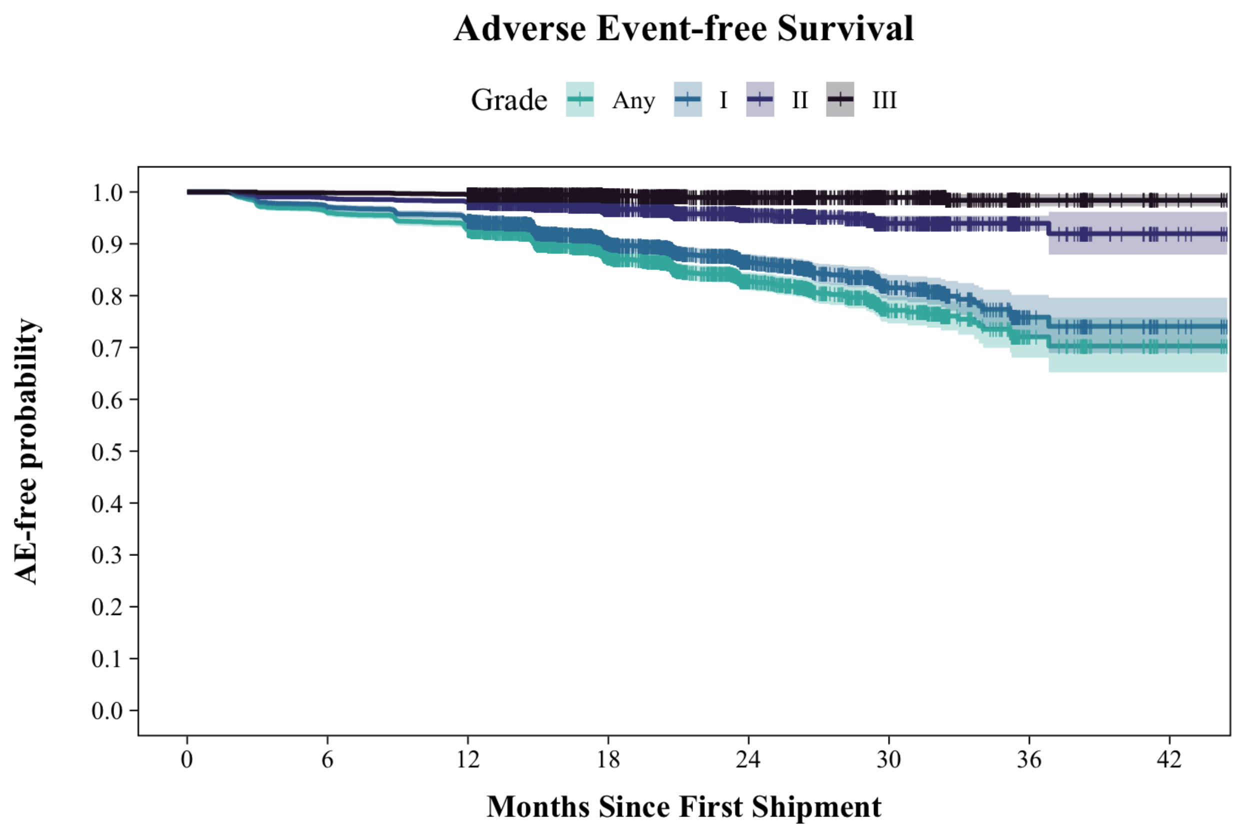

Number at risk

	0	6	12	18	24	30	36	42
Any	2897	2781	2680	1426	669	252	43	6
I	2897	2812	2731	1460	688	262	45	7
II	2897	2860	2834	1524	735	282	50	7
III	2897	2892	2878	1564	752	294	51	6

Cumulative number of events

	0	6	12	18	24	30	36	42
Any	0	116	212	331	387	417	426	427
I	0	85	161	259	302	328	338	339
II	0	37	58	86	99	107	107	108
III	0	5	14	19	23	23	24	24

Cumulative number of censoring

	0	6	12	18	24	30	36	42
Any	0	0	5	1140	1841	2228	2428	2464
I	0	0	5	1178	1907	2307	2514	2551
II	0	0	5	1287	2063	2508	2740	2782
III	0	0	5	1314	2122	2580	2822	2867

Supplemental Figure 5. Time-to-Adverse Event Analysis by CTCAE grade.
