# Supplementary material for: Real-world outcomes of personalized sublingual immunotherapy for environmental allergies delivered through a telemedicine platform: a retrospective longitudinal cohort study
Source: Front Allergy. 2026 Jun 10;7:1865860. doi: 10.3389/falgy.2026.1865860 (PMC13290930; doi:10.3389/falgy.2026.1865860)
Supplement: Supplementary file 8 [file Image6.pdf]

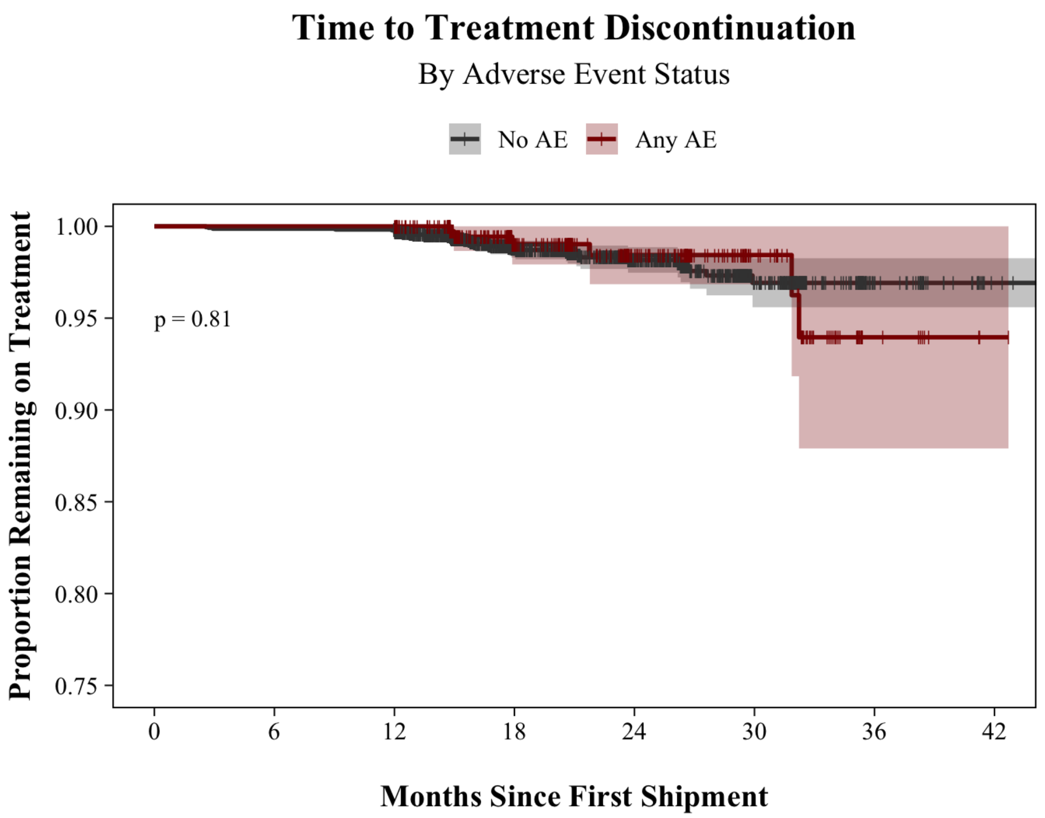

Number at risk

	0	6	12	18	24	30	36	42
No AE	2470	2467	2465	1326	628	240	42	6
Any AE	427	427	427	237	126	54	9	1

Cumulative number of events

	0	6	12	18	24	30	36	42
No AE	0	3	5	25	31	36	36	36
Any AE	0	0	0	3	4	4	6	6

Cumulative number of censoring

	0	6	12	18	24	30	36	42
No AE	0	0	0	1119	1811	2194	2392	2428
Any AE	0	0	0	187	297	369	412	420

Supplemental Figure 6. Time to first self-reported treatment discontinuation, stratified by reporting of any adverse events. P-value is computed from log-rank test comparing survival curves.
